# Tetanus

**Published:** 1916-02

**Authors:** Edward A. Sharp

**Affiliations:** Buffalo, N. Y.


### BUFFALO MEDICAL JOURNAL

Yearly Volume 71. FEBRUARY, 1916 No. 7

### ORIGINAL ARTICLES

The right is reserved to decline papers not dealing with practical medical and surgical subjects, and such as might offend or fail to interest readers. Contributors are solely responsible for opinions, methods of expression and revision of proof.

### Tetanus. With a Report of Two Cases of Sub-Acute Tetanus Followed by Recovery.

By EDWARD A. SHARP, Buffalo, N. Y.

Tetanus has recently become a prominent subject in medical literature, and the large list of drugs advocated in the treatment indicates the unsatisfactory state of the therapeutics of this disease. The old adage that "An ounce of prevention is worth a pound of cure" applies nowhere more strikingly than in tetanus.

The use of the tetanus antitoxin as a prophylactic, given within the first day or two following an injury, is fairly certain in preventing the onset of symptoms, especially if the dose is repeated and proper surgical measures are directed toward the wound.

The situation is very different, however, after the symptoms have developed, and, with all the refinements of modern therapeutics, the one factor most influencing the prognosis is the length of the incubation period. Comby summarizes these well-known facts briefly as follows: "There is an inverse relation between the duration of the incubation and the gravity of the symptoms. With a long incubation the evolution is slow and the prognosis benign; with a short incubation period the evolution is rapid and the prognosis almost invariably fatal."

These facts have been confirmed by the few cases which have been admitted to the Ernest Wende Hospital during the years 1913 to 1915. Of the six cases which have been under treatment all have had a short incubation period, from three to nine days, and, in all, the symptoms were very severe and the results fatal. On the contrary, two cases which I have had under observation during the past year, having a long incubation period—one of two weeks and the other of four weeks —have recovered.

Here, again, the well-known facts, as expressed recently by Nigay, have been observed, "that when the first symptoms of tetanus appear within ten days after the injury the chances of recovery are greatly reduced, and that tetanus is fatal when the incubation period is less than eight days. On the contrary, when the symptoms appear after a long incubation—more than fifteen days—spontaneous recovery is the rule."

Based on the length of the incubation period tetanus is usually classed as acute, when the period is less than ten days, and sub-acute or chronic when the incubation is over ten days.

As the specific cause of tetanus is an anaerobic bacillus, injuries of a certain class are more liable than others to develop tetanus. The dense connective tissue and fascia of the hands and feet make injuries to these members peculiarly susceptible, especially if contaminated with street or stable dirt. While deep, penetrating or crushing wounds are more liable to produce the conditions favorable for infection, surface scratches which become sealed over are also liable, and in a few instances no source of infection could be found. The six acute cases at the Ernest Wende Hospital were of the former type, while one of the sub-acute cases had merely a contusion of the shoulder with slight surface redness as the only ascertainable source of infection.

Injuries received on the battle-field are particularly liable to infection, and reports indicate that the prophylactic use of antitoxin in the present European War is almost universal and the results satisfactory. One report speaks of the highly cultivated soil of a certain district in France as "the tetaniferous region of Vareddes," where every soldier injured developed tetanus unless the prophylactic dose of antitoxin was given immediately after the injury.

The great difficulty in the treatment of tetanus is the lack of a satisfactory method to combat the infection after it has once started. The serum treatment, to be effective, must be given early and in large and repeated doses. If the antitoxin has not been given as a prophylactic within the first twenty-four hours after an injury it should be given on the very first appearance of symptoms. The difficulty appears to be in recognizing the earliest symptoms indicating the onset of tetanus. In all of the six cases mentioned above, the first symptoms which definitely decided the diagnosis and resulted in the case being sent to the hospital were some form of muscle spasm or rigidity. On inquiry it was found that symptoms had been present for twelve to twenty-four hours preceding these visible muscle spasms. In one case, a boy of thirteen years old, said that the day before coming to the hospital, i. e., on the sixth day after a penetrating wound of the foot, he had experienced a sensation of a frequent desire to bite the teeth together—a temporary muscle hypertonus but completely under voluntary control. The next day this became a definite trismus and beyond voluntary control, and the locked jaw definitely established the diagnosis. In another case, a woman thirty years old, had pains in the head and neck, and a slight stiffness and soreness in the throat on the third day after infection. Two days later the jaw became tense and difficult to open, and on the seventh day after infection she was admitted to the hospital.

In a male patient, thirty-five years old, the first symptoms were a slight stiffness in the neck and a sensation of soreness in the throat. Later, temporary spasms of the jaw muscles with difficulty in opening the mouth, coming on eight days after a crushing injury of the left foot, which was amputated within three hours after the original injury.

Occasionally the first symptoms are localized to the part or extremity injured, and later become generalized. Localized tetanus is rare, unless we except the type localized to the cranial motor nerves, called "head-tetanus," and which appears to have a more favorable prognosis than the other types of acute tetanus.

In a boy ten years old, with an infection of the jaw, the first symptoms were a stiffness of the throat and some soreness beginning on the fourth day. The following day severe trismus developed, and the muscle spasm was localized strongly on the right side of the face, while the left face showed partial paralysis, and the mouth was drawn to the right. Later the extremities became involved in severe tetanic convulsions, and a marked opisthotonos occurred as a terminal phenomenon.

Aside from the earliest symptoms, the further clinical manifestations of the disease were the usual and classical symptoms such as headache, stiffness of the neck, face and jaw, with difficulty in swallowing. Convulsions recurred at frequent intervals and sometimes were of considerable duration. At times clonic jerkings of the extremities occurred in place of the tonic rigidity. Cyanosis was a marked symptom in the cases with prolonged tonic spasm, due to involvement of the diaphragm. Profuse perspiration and excessive thirst was a marked symptom in all the cases. Consciousness was retained nearly to the end, except in the one case in which magnesium sulfate was given intraspinally.

The medical treatment of the hospital cases was carried out with the assistance of Dr. Harry Mead, of the consulting staff; the surgical treatment by the surgical staff of the hospital.

Tetanus antitoxin was given in every case. In two instances this was given intraspinally, intravenously and intramuscularly; in one the antitoxin was given intravenously and intramuscularly,. and magnesium sulfate solution given intraspinally; in the other three cases the antitoxin was given intraspinally and intramuscularly, and the Baccelli treatment by hypodermic injection of carbolic acid solution was employed.

The usual dose of antitoxin was 5000 units injected by lumbar puncture into the dural sack; and 10,000 units for intravenous and intramuscular use. This dosage was usually repeated in twenty-four hours.

In conjunction with the specific antitoxin treatment, symptomatic treatment by the use of chloral, morphine, etc., was employed.

Chloral may be given in large doses and the tetanus cases appear to tolerate the drug very well. Some writers have reported using 10.00 to 20.00 grams of the drug daily—sufficient to maintain the patient in a constant stupor, although none of our cases received such massive doses. The usual dose was 10 to 15 grains every four hours.

The Baccelli treatment consists in giving subcutaneous injections of a 3% carbolic solution 5 to 6 c. c. daily in divided doses. It was of no particular service in any of the acute cases.

The use of magnesium sulphate as recommended by Kocher was employed in one case. 6.0 c. c. of a 25% solution was injected into the spinal canal after withdrawal of 30 c. c. of clear spinal fluid. This produced complete relaxation of the severe muscle spasms for 36 hours preceding death. Magnesium sulphate has also been used subcutaneously in a 10 or 20% solution, in doses of 1.5 grams per kilogram of body weight. Like the intraspinal method this produces deep sleep and relaxation of all voluntary muscles.

The danger from the magnesium sulphate solution by either method of administration, is the arrest or embarrassment of respiration, and this may have contributed to the fatal issue in one of our cases.

In view of this danger Zuelzer recommends magnesium glycerophosphate as more effectual and less toxic than the sul phate. 10.0 c. c. of a 25% solution given subcutaneously every three or four hours will keep the convulsions under control.

Ashhurst and John recommend the intra-neural injection of antitoxin and infiltrations of the tissues about the site of the injury, in addition to the use of the antitoxin intravenously and intraspinally. The rationale of this method is based on the fact that the toxin travels along the sheath of the motor nerves and the antitoxin is brought in close relation with the toxin as it travels upward to the spinal cord. It may thus destroy the toxin before it reaches the central nervous system, and prevent the toxin from becoming fixed in the nervous structures.

Kempf employs a somewhat similar method by blocking, with antitoxin, the motor nerves leading to the infected area, and draining the nerve sheath to prevent the central extension of the toxin.

Neither of these latter methods have been employed in our cases, but from the unfavorable experience with other methods this would seem to deserve a trial.

It is to be noted, however, that Kempf's two cases were the sub-acute type in which the tetanus developed after an incubation period of 18 or 20 days, which, in our experience, are favorable under almost any form of symptomatic treatment.

Of interest in this connection are the two cases of sub-acute tetanus which recovered.

Case 1. A young lady, 23 years old, in February, 1915, ran a splinter in right foot. Suppuration occurred and she was lame for ten days, and applied flaxseed poultices to the wound. Two weeks after the injury painful cramps developed in the jaw, and the mouth could not be opened. The extremities became rigid in extensor spasm; the face had a peculiar and stiff sensation, and the muscles were drawn up and the face immovable. The tonic extensor spasms in the extremities continued almost constantly for nearly four weeks, after which time they occurred only periodically and of a few minutes duration. Any sudden jar or noise would bring on an attack. The patient was confined to bed for eight weeks.

About the middle of April the spasms ceased spontaneously, but the left knee was stiff after getting up from bed, and there was a painful rigidity of the spine preventing anterior flexion. In June the stiffness of the spine was quite marked, and there was tenderness over the entire lumbar region and at the fourth dorsal spinous process. A radiogram made by Dr. Koenig showed some irregularity of the body of the fourth dorsal vertebra. There was also some limitation of movements in the right shoulder at this time. During the summer the rigidity of the lumbar portion of the verebral column improved, and the spondylitis at the fourth dorsal vertebra became less marked.

The vertebral symptoms in this case were probably due to the severe and persistent muscle spasm combined with a toxic arthritis, producing a form of traumatic spondylitis.

Tetanus antitoxin was not given, and during the prolonged period of muscular rigidity the treatment was merely symptomatic. There [illegible] from the tetanus

Case II. [illegible] fell [illegible]
a cellar [illegible]
The sho[illegible]

only a slight redness ,probably a skin abrasion, noticed. She had no other injury from this time until the onset of symptoms of tetanus four weeks later. She began to have a sore throat, but on June 25th was able to go to a picnic, and while there she developed a spasm of the muscles of the jaw and was unable to eat but could take liquids. Two days later the trismus became severe and persistent. Tonic spasms of the muscles of the trunk and extremities occurred eight or ten times daily.

On June 29th, 1915, the child was admitted to the Erie County Hospital on the service of Dr. J. S. Otto. During the first two weeks in the hospital there were numerous tetanic spasms lasting from a few minutes to a half hour or longer, and the trismus was very severe and persistent.

One of these convulsive attacks, observed on July 13th, showed the muscles of the face hard and fixed, with the corners of the mouth drawn down, producing the typical "sardonic grin." The jaw was firmly closed and the muscle spasm could not be relaxed, although the child was able to answer questions in monosyllables. The tonic spasm involved the upper extremities, although the child was able to move the arms a little and appeared to gain some relief from the painful spasms by tightly grasping her mother's hands. The proximal muscles were more involved than the distal, and movements at the wrist and fingers were retained. The abdominal muscles were very tense. The lower extremities were held rigidly in extension at the knees and ankles. By exercising slow, strong, passive movements the extensor spasm of the knees could be overcome, and this gave the child some relief. With the knees in the semi-flexed position the knee-jerks were found to be present and not exaggerated. The spasm continued for about ten minutes and then relaxed. The extremities could then be moved about freely and the head could be flexed on the chest, although during the spasm it was markedly retracted. The trismus, however, did not relax during the interval. Spasms of this character were recurring at frequent intervals and were brought on by any noise or excitement.

At this stage of the disease, i. e., 18 days after the onset of the first symptoms and forty-eight days after the injury, the Baccelli treatment was started. The child was given 6 to 8 drops of a 2% phenol solution hypodermically every three hours, and ten grains of chloral hydrate in starch-water enemata every four hours. The following day there was only one convulsion and that occurred during the excitement of a visit from the physician. Sleep was produced and the child kept a drowsy conditio[illegible] [illegible]nol treatment [illegible]ontinued [illegible]y. After [illegible] [illegible]e child [illegible] valescence there was marked stiffness of the vertebral column with pain on movement, and the child experienced some difficulty in using the lower extremities when allowed to walk.

A spondylitis very similar to that observed in the first case was present up to the time the child left the hospital, although showing gradual improvement.

The antitoxin was not used in either of these sub-acute cases. In each case the infection had probably developed its own antitoxin in sufficient amount to protect the patients, and they were in process of spontaneous recovery at the time they came under observation.

In case No. II. the Baccelli treatment cut short the muscle spasms and shortened the course considerably as compared with the first case in which no such treatment was given and the spasms continued for about four weeks.

The immediate after-effects of each of these cases was a form of traumatic spondylitis which gradually recovered.

481 Franklin Street.

---

**Resection of the Stomach.** Professor H. von Haberer (Arch. f. Klin. Chirurg., Bd. 106, Hft. 3) has performed 183 resections of the stomach, 60 for carcinoma and 123 for ulcer. In 54 of the 60 cases of cancer the stomach alone was resected with an operative mortality of 22 per cent., 5 patients surviving four to nine and one-half years. In 6 cases a gastro-colic resection was done with 5 operative deaths. Of the 123 cases of ulcer 8 died after the modified Billroth operation and 3 after transverse resection. There were 66 cases in which the later results could be determined, and of these 61 were in complete health three months to four years after operation. Resection is particularly indicated for cancer and even in apparently inoperable cases permanent results are occasionally obtained. The only contraindications are demonstrable metastases in other organs, multiple peritoneal metastases, and infiltrating growths involving the entire organ. Large size of the tumor, adhesions, local glandular metastases, even of glands in the pancreas, if removable, do not constitute positive contraindications. For cancer the method of Billroth II is the most rational. Both this method and transverse resection are vastly superior to gastro-enterostomy in the treatment of gastric ulcer.—Int. Jour. of Sur.

---

**Glonoin.** Dr. H. M. Farr, Mt. Pleasant, Ia., says a granule of glonoin will relieve the sense of oppression or constriction of the chest, not amounting to actual pain, which often causes restlessness and insomnia. He has found that a single granule dissolved on the tongue will relieve this sensation and that sleep will follow almost immediately.

